# Application of Convolutional Neural Network in Motor Bearing Fault Diagnosis

**DOI:** 10.1155/2022/9231305

**Published:** 2022-08-28

**Authors:** Shuiqin Zhou, Lepeng Lin, Chu Chen, Wenbin Pan, Xiaochun Lou

**Affiliations:** Fair Friend Institute of Intelligent Manufacturing, Hangzhou Vocational and Technical College, Hangzhou 310018, China

## Abstract

In the field of mechanical and electrical equipment, the motor rolling bearing is a workpiece that is extremely prone to damage and failure. However, the traditional fault diagnosis methods cannot keep up with the development pace of the times because they need complex manual pretreatment or the support of specific expert experience and knowledge. As a rising star, the data-driven fault diagnosis methods are increasingly favored by scholars and experts at home and abroad. The convolutional neural network has been widely used because of its powerful feature extraction ability for all kinds of complex information and its outstanding research results in image processing, target tracking, target diagnosis, time-frequency analysis, and other scenes. Therefore, this paper introduces a convolutional neural network and applies it to motor-bearing fault diagnosis. Aiming at the shortcomings of fault signal and convolutional neural network, a large-scale maximum pooling strategy is proposed and optimized by wavelet transform to improve the fault diagnosis efficiency of motor bearing under high-voltage operation. Compared with other machine learning algorithms, the convolution neural network fault diagnosis model constructed in this paper not only has high accuracy (up to 0.9871) and low error (only 0.032) but also is simple to use. It provides a new way for motor bearing fault diagnosis and has very important economic and social value.

## 1. Introduction

With the development of the economy, mechanical equipment is developing towards large-scale and integration, and the structure is becoming more and more complex, such as high-speed railway, aviation aircraft, nuclear power plant, and so on. These equipment put forward high requirements for reliability. If mechanical faults cannot be diagnosed in time, it may lead to machine shutdown, economic losses, and even accidents [1, 2]. Therefore, the research on mechanical fault diagnosis is of great significance.

Rolling bearing is an important part of rotating machinery. It plays an important role in supporting the high-precision rotation of the mechanical rotating body. It is a key component prone to damage [3]. Different electric rotating machinery and equipment often work in various complex working environments, and the uncertain change of working environment can easily lead to different types of damage to rolling bearing, such as high temperature, high heat, high speed, overload load, and so on. If these factors are not controlled in time, they will greatly affect the working life of bearing [4]. As the key parts of most rotating machinery and equipment, once the rolling bearing fails, the replacement of maintenance goods is not timely, which is likely to lead to the paralysis of the whole power unit and even the scrapping of the equipment, resulting in major accident casualties and huge loss of economic property [5, 6].

In recent years, with the rise of computer technology, in order to more accurately and quantitatively describe the performance indexes of electromechanical equipment, many scholars are committed to the research of information diagnosis technology of motor bearing fault. By introducing dyadic discrete wavelet transform, Chen et al. realized the automatic selection of weight factors in the network and then realized the automatic acquisition of bearing fault characteristics [7]. Tan et al. used the public data set of bearing fault of Case Western Reserve University in the United States for diagnosis. The final results showed that this method performed significantly better than the traditional BP neural network [8–10]. Li et al. proposed a bearing fault diagnosis technology combining CNN and wavelet time-frequency map by using a convolution network for excellent feature extraction and generalization performance of data. Relevant experiments were carried out using fault data sets. The results show that this method can effectively classify bearing fault types [11–13]. Han et al. developed a bearing fault classification model based on a deep neural network (DNN). The feature of this method is to directly extract the features of the original fault data and use it as the feature input of the deep neural network model to realize the diagnosis of fault data [14, 15]. Aiming at the problem of manual acquisition of fault characteristics in traditional fault diagnosis, Lu et al. proposed a bearing fault diagnosis method based on a deep self-coding network and carried out experiments under the network model. The results show that this method can effectively diagnose the fault data [16–18]. David et al. proposed a novel network model LAMSTAR and then used the proposed lamstar model to classify the bearing fault signals. Under this method, the equipment is tested at different speeds, and finally, the fault identification accuracy is more than 96% [19–23].

It can be seen from the above research that at present, the CNN fault diagnosis model has been widely used in motor bearing fault diagnosis, and the accuracy of bearing fault diagnosis has gradually improved with the deepening of research. However, many studies also found that when the machine works, because the load often changes greatly and the speed will change within a certain range, it is difficult for the conventional method to be robust to the load and speed at the same time. In addition, the acquisition of training samples is very difficult, and the weak generalization ability in the case of small samples also limits the development of these fault diagnosis methods. The strategy of weight summation and large-scale maximum pooling is proposed to solve the translation invariance of features and enhance the generalization ability of small samples. Improve the practical application ability of the CNN motor bearing fault diagnosis model in bearing fault diagnosis.

## 2. Basic Theory of CNN

### 2.1. Basic Structure of CNN

**Figure 1:**
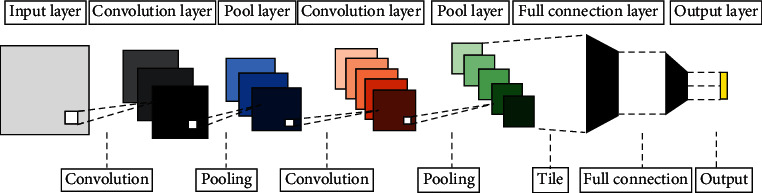
Classic structure of CNN.

The CNN is generally composed of three neural network layers: convolutional layer, pooling layer, and fully connected layer, as well as an output layer (softmax and other classifiers). Its essence is a multilayer perceptron (MLP) neural network. Each layer is composed of multiple two-dimensional plane blocks, and each plane block is composed of multiple independent neural elements, as shown in Figure 1.

#### 2.1.1. Convolutional Layer

The convolutional layer is partially connected with the neurons of the upper layer through the local receptive field. The neurons in the same local receptive field are associated with the corresponding pixels in the image area with fixed two-dimensional plane coding information, forcing the neurons to extract local features. Many groups of different neurons are distributed at each position of each layer, and each group of neurons has a set of input weights. These weights are associated with the neurons in the rectangular block of the previous neural network, that is, shared weights, which reduces the number of weights and the complexity of the network model. Convolutional layer plays an important role in feature extraction in CNN. The observation features obtained by the local sensing domain method are independent of translation, scaling, and rotation. Its weight-sharing structure reduces the number of weights and further reduces the complexity of the network model.

#### 2.1.2. Pooling Layer

The pooling layer is a feature mapping layer. Different continuous ranges in the convolution feature map are selected as the pooling area, and then the maximum or average value of the feature is taken as the feature of the pooling area, so as to reduce the dimension of the feature vector, realize the local average and sampling, and reduce the sensitivity of the feature mapping output to translation, rotation, scaling, and other forms of transformation. The pooling layer usually follows the convolutional layer, which forms a structure of twice feature extraction, so that the network has good distortion tolerance in the identification of input samples.

#### 2.1.3. Fully Connected Layer

The fully connected layer is a structure in which the neurons in this layer and the neurons in the upper layer are connected in pairs, but the neurons in this layer are not connected. It is equivalent to the hidden layer in the multilayer perceptron (MLP). The local feature information is used as the input of the output layer (softmax and other classifiers), and then the convolutional layer is no longer connected. Because after passing through the fully connected layer, the image feature has been reduced from two-dimensional information to one-dimensional information, and the two-dimensional convolution operation cannot be carried out.

#### 2.1.4. Output Layer (Softmax Classifier)

Softmax classifier is the generalization of the logistic regression model in multicategory classification. It can predict the possibility (the number of types of sample labels), but here, it is required that the label of each sample must be unique. If it is a multilabel sample, the softmax model is not applicable. Assuming that the input feature is marked as *x*^(*i*)^ and the sample label is marked as *y*^(*i*)^, which constitutes the training set *S* = {(*x*^(1)^, *y*^(1)^), (*x*^(2)^, *y*^(2)^),…, (*x*^(*m*)^, *y*^(*m*)^)} of supervised learning at the classification level, the forms of hypothesis function *h*_*θ*_(*x*) and logistic regression cost function are as follows:

(1) $$\begin{array}{c} h_{\theta }\left(x\right)=\frac{1}{\sum_{j=1}^{k}e^{\theta_{j}^{T}x^{\left(i\right)}}}\left[\begin{array}{c} e^{\theta_{1}^{T}x^{\left(i\right)}}\\ e^{\theta_{2}^{T}x^{\left(i\right)}}\\ \vdots \\ e^{\theta_{k}^{T}x^{\left(i\right)}} \end{array}\right], \end{array}$$

where *θ*_1_, *θ*_2_,…, *θ*_*k*_ is the learnable parameter of the model and 1/∑_*j*=1_^*k*^*e*^*θ*_*j*_^*T*^*x*^(*i*)^^ is the normalized term.

(2) $$\begin{array}{c} J\left(\theta \right)=-\frac{1}{m}\left[\sum_{i=1}^{m}\sum_{j=1}^{k}1\left\{y^{\left(i\right)}=j\right\}\ln\frac{e^{\theta_{j}^{T}x^{\left(i\right)}}}{\sum_{l=1}^{k}e^{\theta_{l}^{T}x^{\left(i\right)}}}\right], \end{array}$$

where 1{*·*} is an indicative function, that is, when the value in braces is true, the result of the function is 1; otherwise, the result is 0.

The optimal solution of softmax's cost function is usually solved by iterative algorithms such as random gradient descent method (SGD), Newton method, and quasi-Newton method (L-BFGS).

### 2.2. Error Propagation of CNN

The convolution neural network adopts error backpropagation and then is optimized by gradient descent method (or conjugate gradient method, L-BFGS method). Residual *δ* is defined as the influence of the node on the error of the final output value, so residual *δL* of output layer *L* is

(3) $$\begin{array}{c} \delta^{L}=f^{\prime }\left(z^{L}\right)⊙\left(y^{n}-o^{n}\right), \end{array}$$

where ⊙ represents point-by-point product. Therefore, the weight and bias gradient of the output layer, respectively, are

(4) $$\begin{array}{c} \left\{\begin{array}{c} \frac{\partial E}{\partial W^{l}}=x^{l-1}{\left(\delta^{l}\right)}^{T},\\ \frac{\partial E}{\partial b}=\delta^{L}. \end{array}\right) \end{array}$$

The convolutional layer is followed by a lower sampling layer, so the upper sampling is required first when calculating its residual.

(5) $$\begin{array}{c} \delta_{j}^{l}=\beta_{j}^{l+1}\left(f^{\prime }\left(z_{j}^{l}\right)⊙\text{up}\left(\delta_{j}^{l+1}\right)\right), \end{array}$$

where up(*·*) indicates upsampling operation. For maximum pooling, copy the residual in layer *l*+1 to the position corresponding to the maximum in layer *l* and fill the positions of other elements with zero. Summing all elements in the error signal *δ*_*j*_^*l*^ is the gradient of bias in the convolutional layer.

(6) $$\begin{array}{c} \frac{\partial E}{\partial b_{j}}=\sum_{u}{\left(\delta_{j}^{l}\right)}_{u}. \end{array}$$

Considering that the convolution kernel *k*_*ij*_^*l*^ is connected with many shared weights, the region corresponding to the convolution kernel *k*_*ij*_^*l*^ in the convolution process needs to be considered when calculating its gradient through backpropagation.

(7) $$\begin{array}{c} \frac{\partial E}{\partial k_{ij}^{l}}=\sum_{u}{\left(\delta_{j}^{l}\right)}_{u}{\left(p_{i}^{l-1}\right)}_{u}, \end{array}$$

where (*p*_*i*_^*l*−1^)_*u*_ represents the area multiplied by convolution kernel *k*_*ij*_^*l*^ in the convolution process in layer *x*_*i*_^*l*−1^.

For the lower sampling layer where the next layer is connected with the convolutional layer, the shared weight needs to be considered when calculating the residual.

(8) $$\begin{array}{c} \delta_{j}^{l}=\sum_{u}{\left(\delta_{j}^{l-1}\right)}_{u}{\left(q_{i}^{l-1}\right)}_{u}, \end{array}$$

where (*q*_*i*_^*l*−1^)_*u*_ represents the weight in the corresponding convolution kernel *k*_*ij*_^*l*^.

After the gradient is calculated by the above method, the convolution kernel, bias, and the fully connected parameters of the last layer are updated to realize the automatic learning of the convolution kernel, so as to complete the expression and recognition of the signal.

## 3. CNN Fault Diagnosis Model

### 3.1. Basic Model of CNN Fault Diagnosis

**Figure 2:**
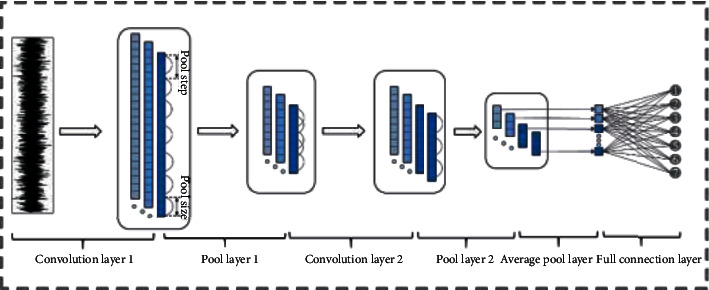
Basic model structure of CNN.

The idea of the basic model structure of CNN in this paper is shown in Figure 2.

### 3.2. Basic Idea of CNN Fault Diagnosis

**Figure 3:**
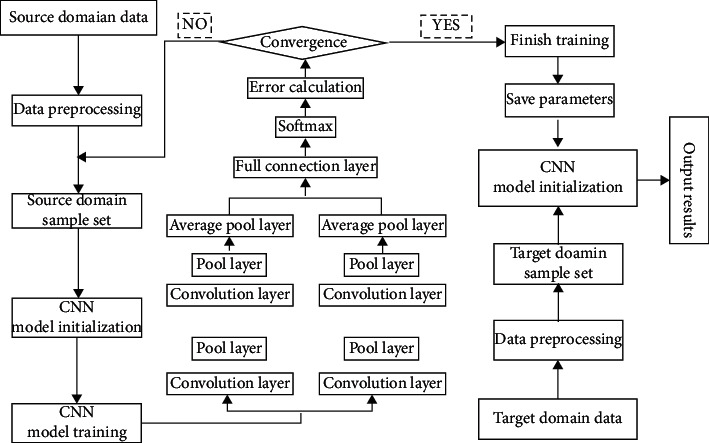
CNN bearing fault diagnosis idea.

The idea of bearing fault diagnosis in this paper is shown in Figure 3.

### 3.3. CNN Fault Diagnosis Model Optimization

CNN's unique topology makes it invariant to feature translation and scaling although CNN has been proved to have good application effects in many fields, such as digital recognition, face recognition, voice recognition, pedestrian diagnosis, and so on. However, there are great differences between motor bearing fault diagnosis and the above problems, and the traditional CNN model is difficult to be effectively applied. Therefore, in the application of motor bearing fault diagnosis, in order to accurately diagnose the rolling bearing fault and reduce the impact of rolling bearing fault on equipment operation, the combination of wavelet packet transform analysis and convolutional neural network is proposed to optimize the motor rolling bearing fault diagnosis model. The optimization steps are as follows:

**Step 1 .**

**Figure 4:**
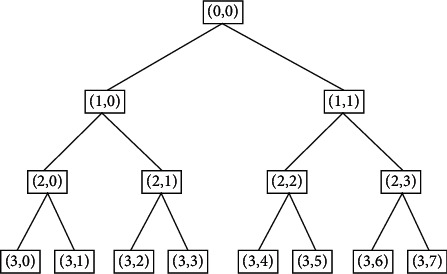
Tree structure of wavelet packet decomposition.

Denoise the bearing vibration signal through wavelet packet transform and analyze the signal in the time domain and the frequency domain by wavelet packet analysis method. The three-layer decomposition process of the wavelet packet is shown in Figure 4.

**Step 2 .**

Extract the eigenvalues of the denoised signal with the eight characteristics of the signal as indicators and diagnose the bearing fault a priori through the time domain and frequency domain information map.

**Step 3 .**

Taking the extracted eigenvalues as samples, the bearing fault category is classified and diagnosed by a convolution neural network model.

## 4. Experimental Process

**Figure 5:**
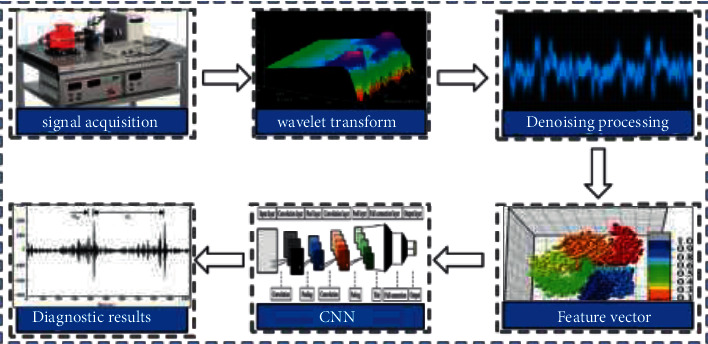
Fault diagnosis flow chart after wavelet transform.

The vibration signal of the rolling bearing is collected through the rolling bearing fault simulation test bed. Firstly, the collected signal is processed by wavelet packet transform, and the noise signal is eliminated to facilitate the subsequent diagnosis. Then extract the fault feature information, input the obtained feature vector into CNN for training and testing, and finally diagnose the fault category through CNN. The specific process is shown in Figure 5.

### 4.1. Data Acquisition

The experimental data come from the comprehensive mechanical fault simulation experimental platform. The experimental platform is shown in the first figure of Figure 5. The platform mainly includes five parts: engine, governor, rotor, shaft, and rolling bearing. The rolling bearing with a fault diameter of 0.3 mm is selected as the experimental object, and the sensor is arranged at the end of the fault bearing.

The experiment collects a variety of fault data at 10 Hz, 20 Hz, and 40 Hz. The data types include the faults of the outer ring, the inner ring, and rolling element and the data of normal bearing. Set the sampling frequency to 1 kHz, take the sampling time for 3 min, and finally pass the data collected by the sensor through MATLAB 2016b ® (The MathWorks, Inc, Natick, MA, USA) software conversion to save the mat file. After that, the signal is effectively cut. According to the sampling frequency, every 1,024 sampling points are selected as a whole fault sample. The four data types under each working condition are 100 samples, except for 300 normal signal samples. Finally, the sum of the four fault type sample data under each working condition is 600. Then the data is preprocessed and divided into two groups: one is used for model training, which is called the training set, and the other group is used for model testing, which is called the test set.

### 4.2. Data Preprocessing

The data preprocessing process mainly is as follows: using data window shift technology to enhance the original time-domain vibration signal and generating training and test sample sets. The “single heat” coding method is used to label the fault type, and then all the data in the training and test set are standardized.

#### 4.2.1. Data Enhancement

**Figure 6:**
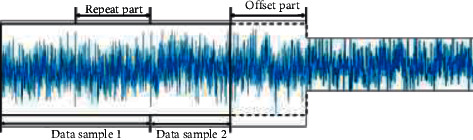
Overlapping sample segmentation.

To realize the high recognition rate of deep learning fault diagnosis, a large number of data samples are needed as support, and the correlation between the fault vibration signal of the rolling bearing cage and the adjacent time series signal is retained. In this paper, a data window shift technique is proposed to segment overlapping samples to achieve data enhancement. The division method is shown in Figure 6. This method can preserve the continuity and periodicity between time series signals, avoid the problem that equal distance sampling is difficult to express all the information characteristics of signals, greatly increase the total number of samples input to network training, and give full play to the powerful learning ability of the model.

As can be seen from Figure 6, if the total length of vibration signal data in fault state is *L*, the length of the sample is *l*, and the vibration signal is divided by a certain offset *m*, the data length of the overlapping part is *l* − *m*.

(1) Number of divisible samples under current signal length *D*:
  (9) $$\begin{array}{c} Ll-mD=\left[\frac{L-l}{m}+1\right], \end{array}$$
where [*·*] is the downward rounding operator.
Expansion multiple of the sample set after data enhancement *β*:
  (10) $$\begin{array}{c} \beta =\frac{l\left(L-l+m\right)}{Lm}. \end{array}$$
(2) Position *x*_*i*_ of the *i* segmented sample in the vibration signal data.
  (11) $$\begin{array}{c} x_{i}=L\left[\left(i-1\right)\times m+1:\left(i-1\right)\times m+l\right],i\in \left[1,D\right], \end{array}$$

where *B* is the split sample, and sample data sets of different sizes can be obtained by setting the corresponding offset *M*.

#### 4.2.2. Data Standardization

In order to improve the convergence speed and accuracy of the model in deep learning, the sample set is usually standardized and preprocessed. The standardized formula is as follows:

(12) $$\begin{array}{c} \bar{x}=\frac{x-x_{\text{mean}}}{x_{std}}. \end{array}$$

Where *x*_mean_ is the mean value of sample data, *x*_*std*_ is the standard deviation of sample data, and *x* is the result after standardization.

At present, formula (12) is one of the most commonly used methods in big data normalization processing. Compared with other data normalization processing formulas, this formula has a better processing effect. Using this formula can also greatly improve the convergence speed and accuracy of the model.

After data standardization, the gradient explosion during model training can be prevented, and the reliability of the results can be guaranteed.

#### 4.2.3. Time-Frequency Analysis Based on Wavelet Transform

**Figure 7:**
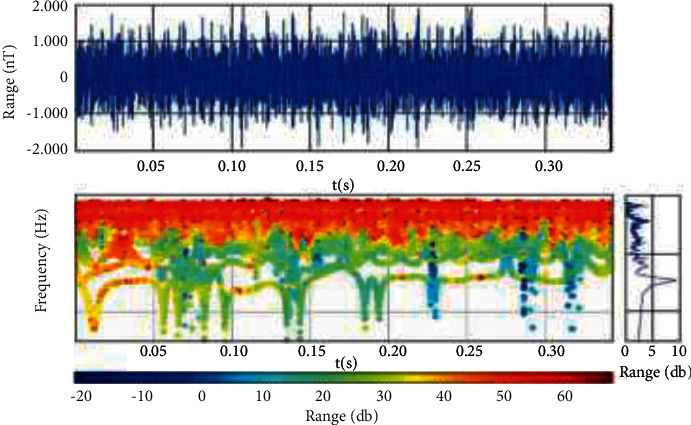
Morlet mother wavelet transform.

In the time-frequency analysis of unstable signals, a wide window is needed to analyze the low-frequency signals in the low-frequency region. On the contrary, a narrow window is needed in the high-frequency region to obtain high-precision amplitude information. However, in the fast Fourier transform, the shape of the window function is fixed. When analyzing the nonstationary signal, there is no adaptability for the signals with different time intervals. Therefore, the fast Fourier transform has some limitations. In order to solve this limitation, the Morlet mother wavelet is introduced into the time-frequency analysis in this paper. Its window area is fixed. It can change the length and height of its window according to the high- and low-frequency characteristics of the signal to adapt to the signal characteristics and ensure the accuracy of time-frequency analysis, its unique multiresolution characteristics, and the advantages of time-frequency localization, which can well meet the needs of practical engineering. The time-frequency analysis results of the Morlet wavelet are shown in Figure 7. It can be seen from the figure that the Morlet wavelet has a relatively concentrated energy distribution of signal, better frequency resolution at high frequency, more accurate time positioning, and slightly better time-frequency focusing. When this kind of time-frequency analysis method is applied, it not only can reduce the burden of time-frequency analysis but also can effectively improve the learning efficiency and diagnosis accuracy of convolutional neural networks.

### 4.3. Establishment of CNN Model Based on Bearing Fault Detection in This Paper

The convolutional layer is a supervised deep learning network, which can extract the fault features in the rolling bearing signal by scanning the convolution kernel on the axis of the image. Usually, a convolutional layer has one or more convolutional nuclei. The neurons of two adjacent convolutional layers are connected with each other. The number of connected neurons is related to the size of the convolution nucleus. By scanning the features, the convolution kernel can take the input data as a matrix, multiply and sum the values in the matrix, and finally superimpose all deviations as follows:

(13) $$\begin{array}{c} Z^{l+1}\left(i,j\right)=\left[Z^{l}\omega^{l+1}\right]\left(i,j\right)+b=\sum_{k=1}^{K_{t}}\sum_{x=1}^{f}\sum_{y=1}^{f}\left[Z_{k}^{l}\left(s_{0}i+x,s_{0}j+y\right)\omega_{k}^{l+1}\left(x,y\right)\right]+b,\\ \left(i,j\right)\in \left\{0,1,\ldots ,L_{l+1}\right\}=\frac{L_{l}+2p_{0}-f}{s_{0}}+1, \end{array}$$

where *b* is the deviation in neurons; *Z*^*l*^ and *Z*^*l*+1^ are the input part and the output part of layer *l* + 1 convolutional layer, respectively; *L*_*l*+1_ is the size of the output part *Z*^*l*+1^; *Z*(*i*, *j*) is the pixel of time-frequency spectrum after wavelet transform; *K* is the number of channels of the map (the time-frequency map used in this paper is an RGB image, and the number of channels is 3); *f*, *s*_0_, and *p*_0_ are the parameters of convolution neural network layer, where *f* is the size of convolution kernel, *s*_0_ is the step size of convolution operation, and *p*_0_ is the size of padding pixels.

**Figure 8:**
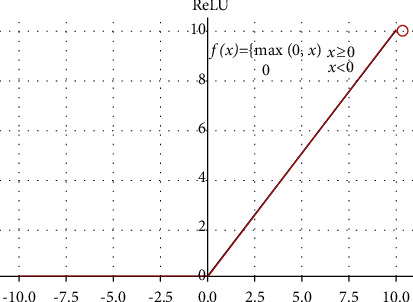
ReLU activation function.

Among them, the activation function used by each convolutional layer is ReLU activation function, and its expression and function image are shown in Figure 8.

After extracting fault features in the convolutional layer, a pooling layer is often needed to filter the extracted fault information and the useless features. The pooling function of the pooling layer is usually preset, which can reduce the dimension of the output of the convolutional layer and make the data compact. This paper selects the maximum pooling layer in the pooling layer, which is used to reduce the estimation mean deviation caused by errors in the training process of the convolutional layer. Generally, the maximum pooling layer is set after the convolutional layer, and the output characteristics of the convolutional layer are maximized by obtaining the maximum value of the feature points. Mathematically, the *l*-th feature map *y*_*n*_^*l*^ of the *n*-th pooling layer can be expressed as follows:

(14) $$\begin{array}{c} y_{n}^{l}=\text{pool}\left(y_{n}^{l},p,s\right), \end{array}$$

where *y*_*n*_^*l*^ is the *n*-th input mapping, that is, the *n*-th input mapping of the previous convolutional layer; pool() is the maximum pool equation; *p* is the maximum pool size; and *s* is the step size of the maximum pool.

**Figure 9:**
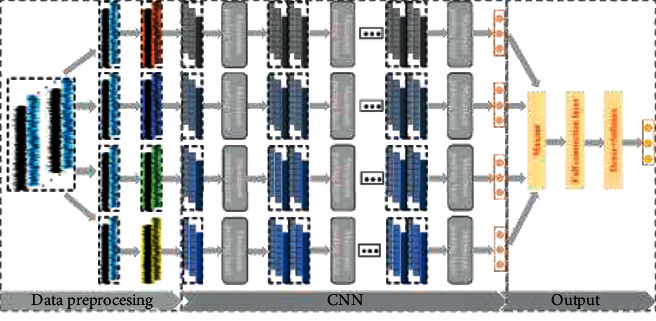
CNN model framework.

The CNN architecture constructed in this paper is shown in Figure 9. In the proposed CNN model, four different convolution paths are used to learn the representation from the input sensor data. Each convolution module is composed of two parts: two-dimensional convolutional layer and maximum pooling layer. These convolutional layers and maximum pooling layers are arranged in parallel, and four different convolution kernel sizes are used to extract the fault features at different speeds. In feature learning, there is no interaction between the four convolution paths, and the fault information is extracted independently from the convolutional layers with different convolution kernel sizes, so as to ensure the integrity of feature extraction. In addition to using different convolution kernel sizes, the four convolution paths are the same in network structure. Each convolution path is constructed by stacking one convolutional layer plus one maximum pooling layer five times. After each convolution path, an attention module is added to learn the weight of each channel, suppress useless feature information, emphasize fault-related information, and then flatten these learned features into a one-dimensional vector through the flatten layer. Finally, the four vectors are combined and input into the classification module for classification.

## 5. Analysis of Experimental Results

### 5.1. Performance Evaluation of Wavelet Transform

**Figure 10:**
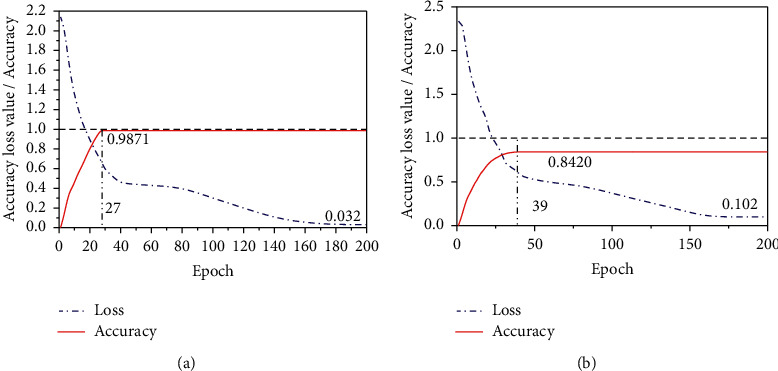
CNN training convergence curve: (a) CNN after wavelet transform and (b) CNN.

After the network is constructed, the classification prediction results are obtained after 200 times of iterative training, as shown in Figure 10.  Figure 10(a) shows the training convergence curve of the CNN after wavelet transform, and Figure 10(b) shows the training convergence curve of the CNN without wavelet transform. Comparing the two figures, it can be found that the network errors of the two models gradually decrease with the increase of epoch. After 27 iterations of the CNN through wavelet transform, the network fully converges. At this time, the network accuracy reaches the best accuracy of 98.71%, and the loss is only 0.032. However, the CNN without wavelet transform needs 40 iterations to converge. At this time, the network accuracy is only 0.8420, but the error is 0.102. It can be seen that compared with the traditional CNN fault detection model, the result of training the network by preprocessing the data through wavelet transform converges faster, and the accuracy is greater than that of training the network without wavelet transform. It shows that the nonstationary original data can be processed through wavelet transform, which reduces the time of network feature extraction and improves the network accuracy.

### 5.2. Model Generalization and Robustness Verification

**Figure 11:**
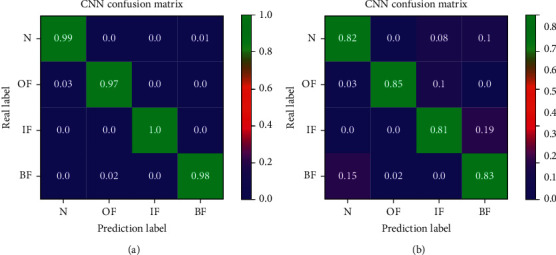
CNN training convergence curve: (a) CNN after wavelet transform and (b) CNN.

It can be seen from Section 4.1 that the CNN model optimized by wavelet transform has high accuracy in detecting the original test set. In order to further verify the generalization ability of the model, build a new test set, input it into the CNN model and count its classification results to obtain the confusion matrix of the test data, and use column summary and row summary to display the accuracy and recall of each class. The results are shown in Figure 11. It can be seen that the CNN model optimized by wavelet transform has a strong generalization ability, and the deep features of the whole original signal can be obtained through less sample learning.

**Figure 12:**
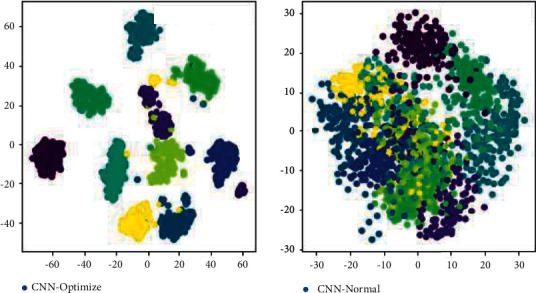
Features visualization for faulty data.

In order to verify the feature extraction ability of the method proposed in this paper, t-SNE dimensionality reduction technology is used to visualize the image features extracted from the traditional CNN model and the last hidden layer of the optimized CNN model proposed in this paper, as shown in Figure 12. The left figure is the CNN fault data feature visualization image after wavelet optimization, and the right figure is the fault data visualization image of the traditional CNN model. It can be seen from Figure 12 that both methods have efficient feature extraction performance for image data, and the fault features representing each fault type have obvious separability in space. However, in terms of spatial clustering, CNN optimized by wavelet transform is obviously better than the traditional CNN model. For example, the features representing the slight damage of the inner ring are distributed in different spatial positions in the feature space of the traditional CNN model, while they are distributed in similar spatial positions in the feature space of the optimized CNN model. The characteristics representing the severe damage of the inner ring also show more dense clustering in the feature space of the optimized CNN model. It further verifies the detection accuracy of the model proposed in this paper for the fault category of rolling bearing.

Because the above verification only tests a group of fault data and cannot represent the detection accuracy of all faults, in order to further verify the feasibility of the method proposed in this paper, the vibration signals and normal vibration signals of 10 different fault positions and different fault degrees at different bearing positions with the measurement speed of 1,797 r/min and the sampling frequency of 12 kHz are selected as the research object, The collected experimental sample data are shown in Table 1.

**Figure 13:**
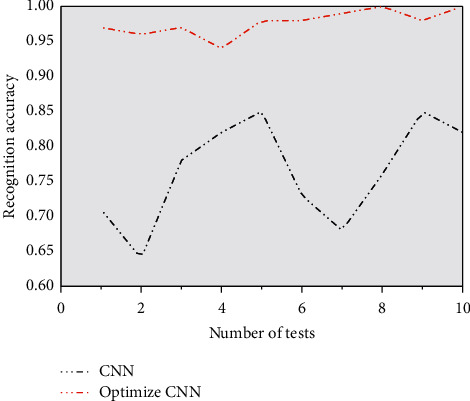
Recognition accuracy of different models.

Figure 13 intuitively shows the fault detection accuracy of the two models in 10 tests. From Figure 10, it can be seen that the CNN algorithm applied to wavelet optimization proposed in this paper has the highest detection accuracy of bearing fault, the smallest numerical fluctuation, and the best stability, which is basically more than 0.95, while the detection accuracy of bearing fault applied to traditional CNN model algorithm is not only lower than that of the optimized model but also fluctuates greatly, and the lowest accuracy is only 0.63, The highest is only 0.85, but the accuracy difference between the two is 34.92%. Thus, the reliability of the optimized CNN model in bearing fault detection is verified again.

## 6. Conclusion

Aiming at the problem that the bearing fault information is difficult to extract completely and the label data samples are insufficient under variable working conditions, this paper proposes a fault detection method based on a convolutional neural network combining wavelet transform and maximum pooling strategy. In this method, the bearing signals at different speeds are used as the input of the fault diagnosis network to integrate the complete fault information. Firstly, the optimized CNN model is constructed by four parallel convolutional neural networks combined with a wavelet transform. Then, after the original data is processed by data enhancement, the time-domain signal is transformed by wavelet transform to obtain the two-dimensional time-frequency spectrum of the fault signal, which is used as the input of the network. Finally, the learned model is used to diagnose and identify the test data. Using the bearing fault data at different speeds to verify the proposed optimized CNN model, it can be concluded that this method has the following advantages:

1. Wavelet transform preprocessing of fault data can reduce the influence of redundant background noise in the data, and when the one-dimensional fault time-domain signal is converted into a two-dimensional fault, the frequency energy spectrum can give better play to the feature extraction function of convolutional neural network.
2. The optimized convolution neural network can select different convolution parameters according to different frequency conversions to extract more complete fault features and highlight fault information. The convolution neural network fault diagnosis model constructed in this paper not only has high accuracy (up to 0.9871) and low error (only 0.032) but also is simple to use, providing a new way for motor bearing fault diagnosis.
3. The data enhancement method can effectively expand the data set and can effectively improve the convergence speed and accuracy of the network while expanding the data set.

## Figures and Tables

**Table 1:** Experimental sample data.

Test number	Detection position	Damage diameter (mm)	Training/validation samples	Test sample
1	Inner	0.3105	1,600/400	400
2	Outer	0.2987	1,600/400	400
3	Roller	0.4136	1,600/400	400
4	Roller	0.2254	1,600/400	400
5	Normal	0.0000	1,600/400	400
6	Roller	0.3512	1,600/400	400
7	Inner	0.3528	1,600/400	400
8	Normal	0.0000	1,600/400	400
9	Outer	0.2911	1,600/400	400
10	Outer	0.3019	1,600/400	400

## Data Availability

The experimental data used to support the findings of this study are included within the article.
